# A Facile Fabrication of a Potentiometric Arrayed Glucose Biosensor Based on Nafion-GOx/GO/AZO

**DOI:** 10.3390/s20040964

**Published:** 2020-02-11

**Authors:** Jung-Chuan Chou, Si-Hong Lin, Tsu-Yang Lai, Po-Yu Kuo, Chih-Hsien Lai, Yu-Hsun Nien, Tzu-Yu Su

**Affiliations:** 1 Graduate School of Electronic Engineering, National Yunlin University of Science and Technology, Douliu 64002, Taiwan; M10613304@yuntech.edu.tw (S.-H.L.); M10713312@yuntech.edu.tw (T.-Y.L.); kuopy@yuntech.edu.tw (P.-Y.K.); chlai@yuntech.edu.tw (C.-H.L.); 2 Graduate School of Chemical and Materials Engineering, National Yunlin University of Science and Technology, Douliu 64002, Taiwan; nienyh@yuntech.edu.tw (Y.-H.N.); M10715021@yuntech.edu.tw (T.-Y.S.)

**Keywords:** glucose, zinc oxide (ZnO), aluminum-doped zinc oxide (AZO), graphene oxide (GO), potentiometric biosensor, arrayed electrodes, glucose

## Abstract

In this study, the potentiometric arrayed glucose biosensors, which were based on zinc oxide (ZnO) or aluminum-doped zinc oxide (AZO) sensing membranes, were fabricated by using screen-printing technology and a sputtering system, and graphene oxide (GO) and Nafion-glucose oxidase (GOx) were used to modify sensing membranes by using the drop-coating method. Next, the material properties were characterized by using a Raman spectrometer, a field-emission scanning electron microscope (FE-SEM), and a scanning probe microscope (SPM). The sensing characteristics of the glucose biosensors were measured by using the voltage–time (V-T) measurement system. Finally, electrochemical impedance spectroscopy (EIS) was conducted to analyze their charge transfer abilities. The results indicated that the average sensitivity of the glucose biosensor based on Nafion-GOx/GO/AZO was apparently higher than that of the glucose biosensor based on Nafion-GOx/GO/ZnO. In addition, the glucose biosensor based on Nafion-GOx/GO/AZO exhibited an excellent average sensitivity of 15.44 mV/mM and linearity of 0.997 over a narrow range of glucose concentration range, a response time of 26 s, a limit of detection (LOD) of 1.89 mM, and good reproducibility. In terms of the reversibility and stability, the hysteresis voltages (V_H_) were 3.96 mV and 2.42 mV. Additionally, the glucose biosensor also showed good anti-inference ability and reproducibility. According to these results, it is demonstrated that AZO is a promising material, which could be used to develop a reliable, simple, and low-cost potentiometric glucose biosensor.

## 1. Introduction

Glucose plays a vital role in numerous physiological processes, and all cells and organs in the human body require glucose as a source of energy in order to function properly [1]. If blood sugar cannot be sufficiently well regulated, an individual will eventually develop type 2 diabetes (T2D). At present, T2D is an incurable chronic disease, but it is preventable and controllable if symptoms are recognized early enough [2]. Worldwide, approximately 422 million people have some type of diabetes, and it is the cause of 3.96 million deaths annually [3]. For this reason, the detection and control of blood sugar levels are crucially important.

Zinc Oxide (ZnO) is an n-type II-VI semiconductor with large exciton binding energy (60 meV) and a wide direct band gap (3.37 eV) [4]. The ZnO nanostructure may have potential as an excellent matrix owing to its ability to enhance signal transduction and to facilitate the immobilization of biomolecules while retaining activity. A matrix with these properties could be used in the development of a biosensor [5]. Enzymes such as glucose oxidase (GOx) have a relatively low isoelectric point (IEP approximately 4.2) [6]. Conversely, ZnO has a high isoelectric point (IEP approximately 9.5) and thus can provide a suitable environment for enzyme immobilization [7]. Recently, Ali et al. [8] developed a potentiometric glucose biosensor by immobilizing GOx onto a ZnO nanowires (NWs)/Ag electrode. They compared the addition of bovine serum albumin (BSA) regarding the growth of ZnO NWs. The biosensor had an outstanding sensitivity of 35 mV/decade over a relatively wide logarithmic concentration range (0.5 to 1000 μM) and a fast response within 4 s. Fulati et al. [9] proposed a potentiometric intracellular glucose biosensor based on a BSA-GOx/ZnO nanoflakes (NFs)/Al microelectrode, which was used to take measurements from human adipocytes and frog oocytes. The results showed a fast response within 4 s, and a sensitivity of 65.2 mV/decade over a wide range of glucose concentrations (500 nM to 10 mM), as well as good values for stability, selectivity, and reproducibility. Wahab et al. [10] studied a glucose biosensor based on ZnO nanorods (NRs) that were deposited on a silver wire with an annealing temperature of 250 °C. The biosensor was able to determine glucose concentration in phosphate-buffered saline (PBS) over a range of 1µM to 10mM and in human serum, and its linearity was 0.98. The aforementioned biosensors demonstrate the enormous potential for ZnO in the potentiometric detection of glucose.

ZnO has been widely studied in the biosensing field and thus many of its properties and applications are well known [8,9,10,11,12,13]. Aluminum-doped zinc oxide (AZO) is a novel material that is composed of ZnO that had aluminum (Al) doped into it. The electrical conductivity of ZnO can be enhanced by doping group III metals such aluminum, a p-type dopant, into it in order to increase the concentration of free holes. Doping can change the characteristics of a material, such as its electron mobility, optical properties, and electrical conductivity, and can improve its high-temperature stability [14,15]. AZO is more sensitive compared to non-doped ZnO, especially with regard to adsorbed species on the surface in a known microenvironment [16]. Besides, relatively little research has been done in potentiometric biosensors applying AZO in recent years, and most studies have investigated the use of AZO in amperometric biosensors [13,17]. Due to the good sensitivity and limit of detection, the amperometric glucose biosensors have come into the picture. However, when a high polarizing voltage is applied, interfering substances may lead to nonspecific signals. Compared to the amperometric biosensors, potentiometric biosensors have an outstanding advantage in selectivity and stability, due to the extra potential that is not required, so they have become more particularly suitable for long-term monitoring [18]. Therefore, we applied AZO to the sensing membranes of potentiometric arrayed glucose biosensors, thereby analyzing whether AZO was more suitable for biosensing than ZnO.

Graphene is a form of carbon consisting of two-dimensional monolayers of carbon atoms arranged in a honeycomb lattice [19]. Since graphene has excellent electrical conductivity, a high volume–surface area ratio, hydrophobicity, and strong mechanical strength [20,21], it is increasingly being used in the development of sensors [20,21,22,23]. Graphene oxide (GO), which belongs to the graphene family, was also selected for investigation in this study. According to the structural model proposed by He et al. [24], GO consists of unoxidized benzene rings and has a hexagonal ring structure. GO could be dissolved and manipulated in aqueous solution owing to the abundant hydroxyl and epoxide groups on the basal planes and the numerous carbonyl and carboxyl groups at the sheet edges [24,25,26]. GO was also able to enhance the charge transfer ability of sensors and biosensors to improve their sensitivity [26] and limit of detection (LOD) [27]. In this study, GO was synthesized using the modified Hummers’ method [28], and it was processed into an aqueous solution in order to modify the sensing membranes of the potentiometric arrayed glucose biosensors.

In this study, we proposed a potentiometric arrayed glucose biosensor based on a terephthalate (PET) substrate, which has many advantages, such as its portability, flexibility, miniaturization of sensors, and low cost [29]. In terms of the fabrication process, the printed silver pattern served as the reference electrodes and conductive wires on a PET substrate via screen-printing technology [30]. Sputtering was used to deposit a metal oxide, i.e., AZO or ZnO, to form sensing membranes. GO was used to modify the sensing membranes, and the GOx was immobilized on the sensing electrodes by the entrapment method to complete the production of the glucose biosensor. Then, we investigated the sensing characteristics of the potentiometric arrayed glucose biosensor based on Nafion-GOx/GO/AZO, which included the average sensitivity, linearity, LOD, response time, reversibility, selectivity, and reproducibility. Further analysis using electrochemical impedance spectroscopy (EIS) was conducted to determine the charge transfer ability of membranes. Finally, the performances of the proposed glucose biosensor were compared with potentiometric glucose biosensors that have been developed in recent years.

## 2. Experimental

### 2.1. Materials

The silver conductive paste was purchased from Advanced Electronic Material Inc. (Tainan, Taiwan). The epoxy (product no. JA643) was purchased from Sil-More Industrial, Ltd. (New Taipei City, Taiwan). Polyethylene terephthalate (PET) was purchased from Perm Top Co., Ltd. (New Taipei City, Taiwan). D-Glucose was purchased from J. T. Baker Co. (New Jersey, USA). The graphite powder was purchased from Alfa Aesar Co. (Massachusetts, USA). Nafion and glucose oxidase (GOx) were purchased from Sigma-Aldrich Co. (Missouri, USA). The AZO (99.99% purity, Al:ZnO = 2 wt%:98 wt%) and ZnO (99.99% purity) targets were purchased from Ultimate Material Technology Co., Ltd. (Hsinchu, Taiwan). The 0.1 M phosphate-buffered saline solution (PBS, pH 7.0) was prepared by mixing the standard solution of potassium phosphate monobasic (KH_2_PO_4_) and potassium phosphate dibasic (K_2_HPO_4_), which were purchased from Katayama Chemical Co., Ltd. (Osaka, Japan). All chemicals used in this study were of analytical grade and used without further purification.

### 2.2. Deposition of Sensing Membranes and Preparation of Electrodes

The PET substrate was cut into an area of 10.5 cm^2^ (3 cm × 3.5 cm) which was cleaned using ethanol and deionized (D.I.) water in an ultrasonic vibrator for 10 min. The silver conductive paste was printed onto the PET substrate, the electrodes and conductive wires were printed using screen-printing technology, and then they were placed in a high-temperature oven at 120 °C for 30 min.

The high-purity circular AZO and ZnO targets were used for deposition of the sensing membranes. ZnO or AZO was sputtered onto the PET substrate by radio frequency (RF) sputtering at 3 mTorr pressure, 60 W power, and with Ar/O_2_ as the reactive gas flowing at 9/1 sccm for 30 min. In terms of encapsulation, the epoxy was printed on the sensors as an insulation layer, and the completed samples were then placed in a high-temperature oven at 120 ºC for 90 min. The aforementioned process produced six defined areas that served as the sensing windows (1.77 mm^2^ per window), and which were able to protect the conductive wires and block an aqueous solution.

### 2.3. Synthesis of Graphene Oxide and Modification of the Sensing Membranes

The modified Hummers’ method [28] was employed to synthesize GO using graphite powder. Graphite and sodium nitrate (NaNO_3_) were mixed (vol. ratio: 1:1) in a sealed glass container, and sulfuric acid (H_2_SO_4_) was added to the mixture. The mixture was stirred in an ice bath at 0–4 °C for 1 h. Then, potassium permanganate (KMnO_4_) was slowly added into the uniform mixture and stirred for 24 h. Subsequently, deionized (D.I.) water was added into the mixture, followed by hydrogen peroxide (H_2_O_2_) to cease the oxidation reaction, and then the mixture was stood for 24 h. Next, the produced precipitate was washed by using a solution of hydrochloric acid (HCl) and D.I. water (vol. ratio: 1:10). Finally, the precipitate was rinsed with D.I. water until it reached pH 7.0, after which it was freeze-dried to remove any excess water. Finally, the brown-colored GO powder was obtained.

In order to modify the sensing membranes, the GO powder was dissolved in D.I. water and placed in an ultrasonic vibrator for 10 min in order to prepare the 0.3 wt% GO solution [31]. Subsequently, 2 μL of GO solution was separated by a micropipette and used to modify the sensing membranes by the drop-coating method. Thereafter, the membranes were left to dry at room temperature.

### 2.4. Immobilization of Nafion-GOx Sensing Membranes

The 10 mg of glucose oxidase powder was added to 1 mL of 0.1 M PBS solution (10 mg/mL in PBS). The glucose oxidase solution and Nafion were uniformly mixed (vol. ratio: 4:3) by using a vortex mixer [31]. Finally, the mixture of Nafion and glucose oxidase was dropped onto the sensing windows to form the glucose sensing membrane, and then the Nafion-GOx sensing membranes were dried at 4 °C for 12 h. The unbound enzyme was removed by rinsing with D.I. water. After the GOx was immobilized on the sensing windows, the potentiometric arrayed glucose biosensor based on Nafion-GOx/GO/ZnO or Nafion-GOx/GO/AZO was fabricated.

**Figure 1:**
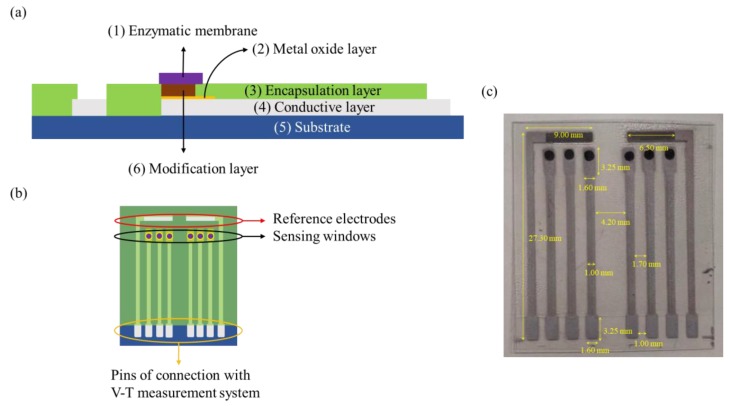
Schematic diagrams of the potentiometric arrayed glucose biosensor: (**a**) cross-section view and (**b**) top view. (**c**) Optical image of the potentiometric arrayed glucose biosensor based on Nafion-GOx/GO/AZO (dimensions annotated in yellow).

The schematic diagram of the potentiometric arrayed glucose biosensor is shown in Figure 1. The function of each layer in Figure 1a is as follows: (1) the enzymatic membrane acts as a biometric layer; (2) the metal oxide layer serves as a matrix; (3) epoxy provides an insulation layer preventing contact with aqueous solution; (4) silver paste is printed on the substrate as reference electrodes and conductive wires; (5) PET is used as a substrate of the flexible arrayed biosensors; (6) GO is used as a modification layer, which is used to enhance the specific surface area of the sensing membranes. In Figure 1b, it can be seen that the biosensor has six sensing windows, two reference electrodes, and eight pins for connection with the V-T measurement system. Figure 1c only shows the optical image of the potentiometric arrayed glucose biosensor based on Nafion-GOx/GO/AZO, which the dimensions annotated in yellow. Since the optical images of ZnO and AZO were almost identical (the membranes were transparent), only one of them is shown in Figure 1.

### 2.5. Voltage–Time Measurement System

**Figure 2:**
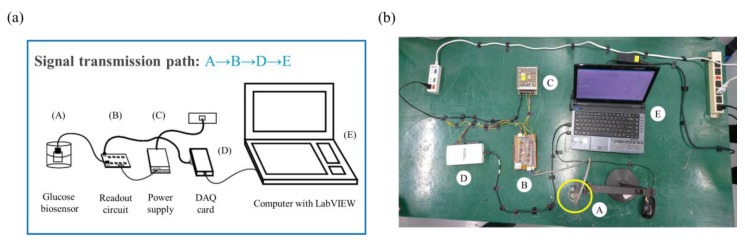
Experimental system diagram of the voltage–time (V-T) measurement system: (**a**) schematic view and (**b**) experimental setup.

**Figure 3:**
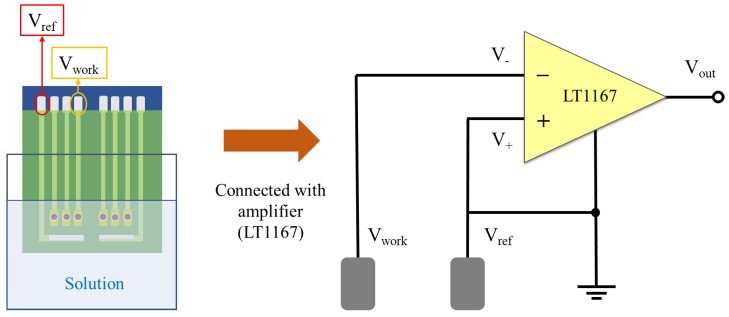
Working schematics of the potentiometric measurement, which utilizes the voltage difference between a working electrode and a reference electrode to perform. LT1167 is the amplifier used in the readout circuit (inset).

The sensing characteristic of the biosensor was measured by a voltage–time (V-T) measurement system [32], which is composed of a power supply, a readout circuit, a data acquisition card (DAQ card) (Model: NI USB-6201, National Instrument Corp. Texas, USA), and the system software (Model: LabVIEW 2011, National Instrument Corp. Texas, USA). The glucose biosensor measured via the V-T measurement system is shown in Figure 2. In this study, due to its high common-mode rejection ratio (CMRR) and high input impedances, LT1167 was determined to be suitable for bio-electronic signals. The readout circuit consists of eight instrumentation amplifiers (INA, LT1167). The output voltage is expressed by Equation (1):

(1) $$V_{\mathrm{out}}\;=\;(V_{+}\;-\;({}_{-}V_{-}))\times \;(1\;+\;\frac{R_{\mathrm{th}}}{R_{G}})=V_{\mathrm{ref}}-V_{\mathrm{work}}=-V_{\mathrm{work}}\;(∵R_{G}=\infty )$$

where V_out_ is the output voltage (INA), V_+_ is the non-inverting input voltage (INA), V_-_ is the inverting input voltage (INA), R_th_ is the internal equivalent resistance, V_G_ is the gain resistance, V_ref_ is the potential of the reference electrode, and the V_work_ is the potential of the working electrode. Herein, because R_G_ is an open circuit (R_G_ = ∞), the voltage gain of INA is 1. According to Equation (1), we can obtain V_out_, which is the potential difference between the working electrode and the reference electrode, i.e., -V_work_ is the response voltage of the biosensor. The working schematics of the potentiometric measurement are shown in Figure 3.

### 2.6. Characterization of Materials

The optical microscope with a light source (Model: VHX-5000 and VH-Z100R, Tokyo, Japan) that was used to observe the morphology of a silver electrode was purchased from Keyence Co., Ltd. The field-emission scanning electron microscope equipped with an energy-dispersive detector (FE-SEM, Model: JSM-6701F, Tokyo, Japan) that was used to investigate the morphology and conduct the elemental analysis was purchased from JEOL Ltd. The Raman spectrometer (Model: iHR550, Tokyo, Japan) with 532 nm laser excitation that was used to characterize carbon materials was purchased from Horiba, Ltd. The scanning probe microscope (SPM, Model: Dimension Icon, Texas, USA) that was used to examine the surface roughness of membranes was purchased from Bruker Corp.

### 2.7. Analysis of Electrochemical Impedance

The electrochemical impedance analysis was characterized by using a potentiostat/galvanostat (Biologic, SP-150, Isère, France), and a three-electrode setup was used with a Pt panel as a counter electrode, an Ag/AgCl reference electrode, and a working electrode. The frequency range was set from 200 kHz to 50 mHz. The amplitude of the sine signal was 10 mV (E_WE_ vs. E_OC_ = 0V). The test solution was a 0.1 M PBS solution (pH 7.0). Other experimental parameters, such as temperature, were kept constant.

## 3. Results and Discussion

### 3.1. Raman Spectroscopy of GO and Morphology of Membrane

**Figure 4:**
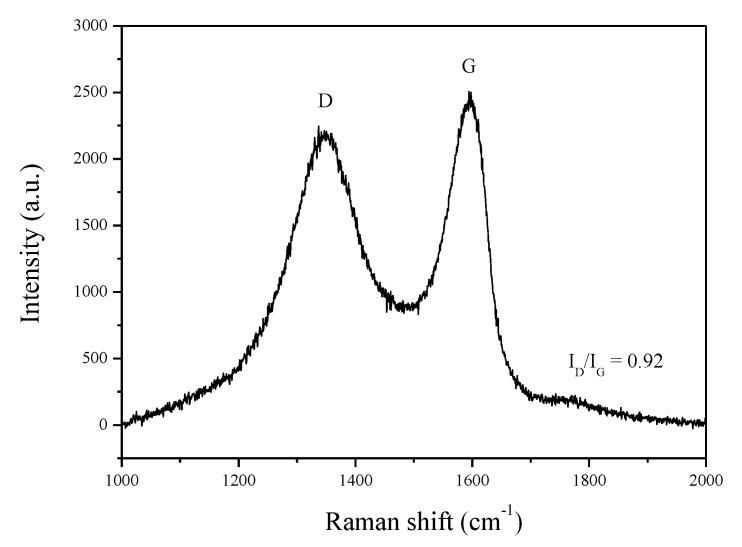
Raman spectrum of graphene oxide (GO).

The synthesized GO was characterized by Raman spectroscopy. Raman spectroscopy is an analytical technique for characterizing the differences between sp^2^ and sp^3^ hybridization in carbon materials [33]. The Raman spectra of GO are characterized by a D band at approximately 1340 cm^−1^ and a G band at approximately 1590 cm^−1^ [34,35]. The D band represents the degree of defects on graphene sheets, resulting from the presence of sp^3^-carbon atoms; the G band is used to evaluate the graphitization degree, originating from in-plane vibrations of sp^2^-carbon atoms [34,35]. The intensity ratio of the D and G bands (I_D_/I_G_) is an index of the degree of defects on graphene [34,35,36]. As shown in Figure 4, the spectrum shows two obvious peaks of D (1347.89 cm^−1^) and G (1595.31 cm^−1^), and the value of I_D_/I_G_ was 0.92. The results indicated the successful synthesis of GO.

**Figure 5:**
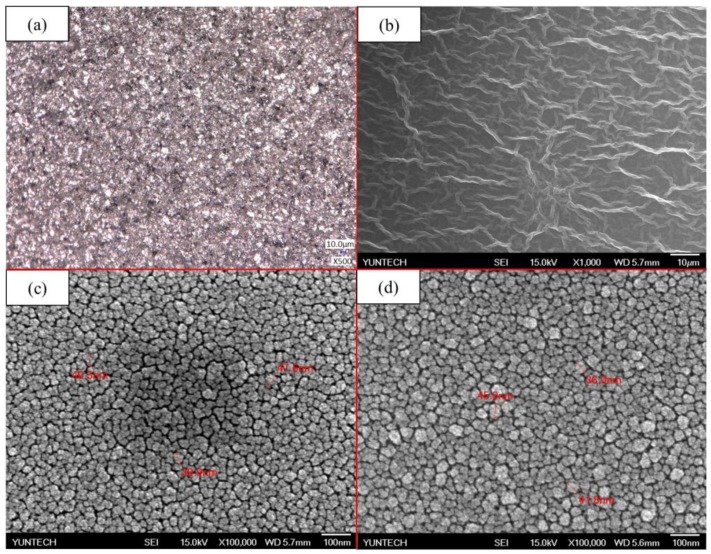
Field-emission scanning electron microscope (FE-SEM) images of different membranes: (**a**) silver, (**b**) GO, (**c**) zinc oxide (ZnO), and (**d**) aluminum-doped zinc oxide (AZO).

**Figure 6:**
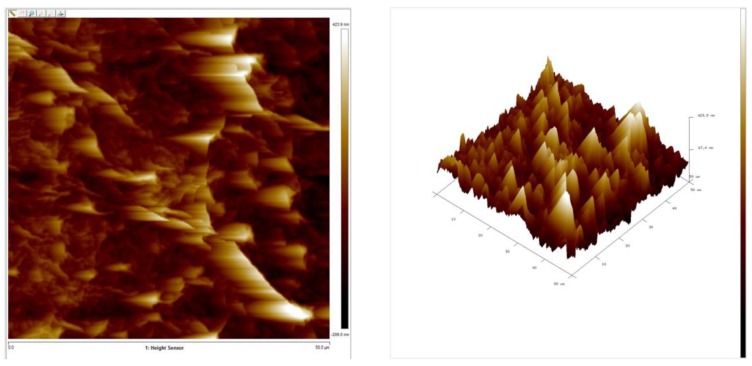
2D and 3D atomic force microscope (AFM) images of GO membrane.

The morphology of materials was characterized by using the FE-SEM and the optical microscope, as shown in Figure 5. From Figure 5a, the surface of a silver electrode, which was used as the conductive layer, was compact. After that, the ZnO and AZO were deposited onto the silver electrode by sputtering. As shown in Figure 5c–d, the ZnO and AZO showed a uniform and compact distribution of nanoparticles. The grain size of both films was nearly the same. In order to compare the differences between the ZnO membrane and the AZO membrane and to determine whether Al was successfully doped into the ZnO membrane, energy-dispersive X-ray spectroscopy (EDX) was used for the qualitative analysis of both membranes. In Figure S1, both EDX spectra all exhibited obvious Si, Pt, O, and Zn peaks. Si and Pt peaks were originated from Si substrates and the preparation of samples, respectively. In Figure S1b, a small Al peak could be found next to the Si peak. This is due to the light doping of the AZO target (Al:ZnO = 2 wt%:98 wt%). The results indicated that the AZO membrane was successfully deposited by sputtering, and there is a difference compared to the ZnO membrane. In Figure 5b, the GO membrane exhibited a highly crumpled surface with jagged wrinkles across the surface. These wrinkles increased the surface roughness of the membrane, which could provide numerous active sites to facilitate the adsorption of ions onto the membrane. Furthermore, the morphology of the GO membrane was further characterized by the 2D and 3D atomic force microscope (AFM) images, as determined by using an SPM. Figure 6 shows an array composed of jagged carbon flakes. The GO membrane exhibited a quite high roughness average (R_a_) of 75 nm and root mean roughness (R_q_) of 99 nm, as determined by the surface roughness analysis. It was reported that the large surface roughness of the electrode was responsible for the good sensing characteristics of a sensor [37]. From these results, the GO membrane with high R_a_ and R_q_ might play an indispensable role in the potentiometric arrayed glucose biosensors.

### 3.2. Average Sensitivity, Linearity, LOD, and Response Time of Potentiometric Arrayed Glucose Biosensors

The sensing mechanism of the potentiometric biosensor follows the Nernst Equation (2) [38]:

(2) $${E\;=\;E}^{0}\;+\;\frac{\mathrm{RT}}{F}{\ln[a}_{H^{+}}{]\;=\;E}^{0}\;-\;2.303\frac{\mathrm{RT}}{F}\;\mathrm{pH}$$

where E is the electromotive force (EMF), E^0^ is the standard potential of the reference electrode, R is the gas constant, T is the temperature in Kelvins, F is the Faraday’s constant, and pH is the pH of the electrolyte. The sensing mechanism of most potentiometric glucose biosensors is based on an enzymatic reaction catalyzed by glucose oxidase (GOx) according to Formulas (3) and (4) [8]:

(3) $$H_{2}O+O_{2}+\mathrm{glucose}\overset{\mathrm{GOx}}{\to }\delta -\mathrm{gluconolactone}+H_{2}O_{2}$$

(4) $$\delta -\mathrm{gluconolactone}\overset{\mathrm{spontaneous}}{\to }{\mathrm{gluconate}}^{-}+H^{+}$$

As shown in Formulas (1) and (2), δ-gluconolactone and hydrogen peroxide were produced after the enzymatic reaction was catalyzed by GOx. The produced δ-gluconolactone spontaneously converted to gluconate ions and hydrogen ions (H^+^). Owing to the local variation of H^+^ in the microsurroundings of a membrane, the different surface potentials are formed further. When the potentiometric arrayed glucose biosensor was measured over a low glucose concentration, the pH of the microsurroundings of a membrane was high; the negative value of E was larger according to Equation (2), and vice versa. Based on Equation (1), the obtained negative value was converted into a positive value via LT1167. This is the sensing mechanism by which the potentiometric glucose arrayed biosensor detects glucose.

**Figure 7:**
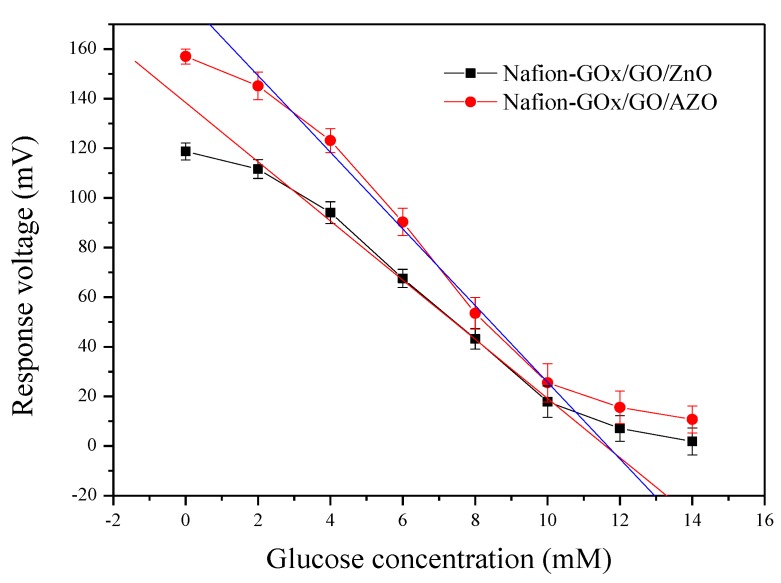
Response curves of the glucose biosensors over glucose concentrations ranging from 0 to 14 mM, for Nafion-GOx/GO/ZnO and Nafion-GOx/GO/AZO. AZO: aluminum-doped zinc oxide, GOx: glucose oxidase.

The response characteristics of the biosensors based on Nafion-GOx/GO/ZnO or Nafion-GOx/GO/AZO were measured in 0.1 M PBS (pH 7.0) solutions with different glucose concentrations, ranging from 0 to 14 mM, by using the V-T measurement system. The average response voltages (mean) and the error bars (standard deviation, SD) were obtained from the response voltages of the six windows (1.77 mm^2^), and then the average sensitivity and linearity were calculated by Origin 7.0. Next, the average sensitivities and linearities of both types of sensors were recorded and analyzed. As shown in Figure 7, the glucose biosensors over a glucose concentration range (2–10 mM) exhibited a linear variation in the response voltages. When the glucose concentration was out of this range, the variation in response voltages was reduced, i.e., the response curve was flatter. Therefore, the linear range of the glucose biosensors was 2–10 mM. In addition, the difference between intervals over the tested glucose concentration range (2–10 mM) were extremely significant, according to the results shown in Table S1. As shown in Figure 7 and Table 1, the glucose biosensor based on Nafion-GOx/GO/AZO performed well, with excellent average sensitivity (15.44 mV/mM), which was markedly higher than that of the glucose biosensor based on Nafion-GOx/GO/ZnO (11.92 mV/mM). The enhancement of the average sensitivity can be attributed to the electrical conductivity of the matrix. By doping a small amount (2 wt%) of Al into ZnO, Al^3+^ ions replaced Zn^2+^ ions in the ZnO lattice, resulting in the lower electrical resistivity of ZnO, thereby improving the sensing characteristics. Apart from this, the level of the response voltages was shifted upward when AZO was used as the matrix of the glucose biosensor.

After determining the average sensitivity of the potentiometric arrayed glucose biosensor based on Nafion-GOx/GO/AZO, the response time and limit of detection (LOD) were then characterized via the V-T measurement system. In order to determine the baseline of the biosensor, the response voltage of the biosensor was measured in pure 0.1 M PBS solutions (pH 7.0) without glucose (measurement times, N = 7); the baseline of the potentiometric glucose biosensor is 157.02 ± 2.01 mV, as shown in Table 1. According to the obtained baseline, the LOD of the potentiometric glucose biosensor was calculated, which was the lowest concentration for the detection of analytes at a specified signal-to-noise ratio (S/N = 3) [39,40]. The LOD was 1.89 mM, which the value was in accordance with the measured results. Finally, the 5 mM glucose was added into a pure PBS solution (pH 7.0) to examine the response time of the potentiometric arrayed glucose biosensor. The response time was the period of time required to achieve 95% of the steady state (containing analytes) from the origin state (without analytes) over the whole concentration range [41]. According to the experimental results, the response time of the potentiometric arrayed glucose biosensor based on Nafion-GOx/GO/AZO was 26 s.

### 3.3. Electrochemical Impedance Spectroscopy

**Figure 8:**
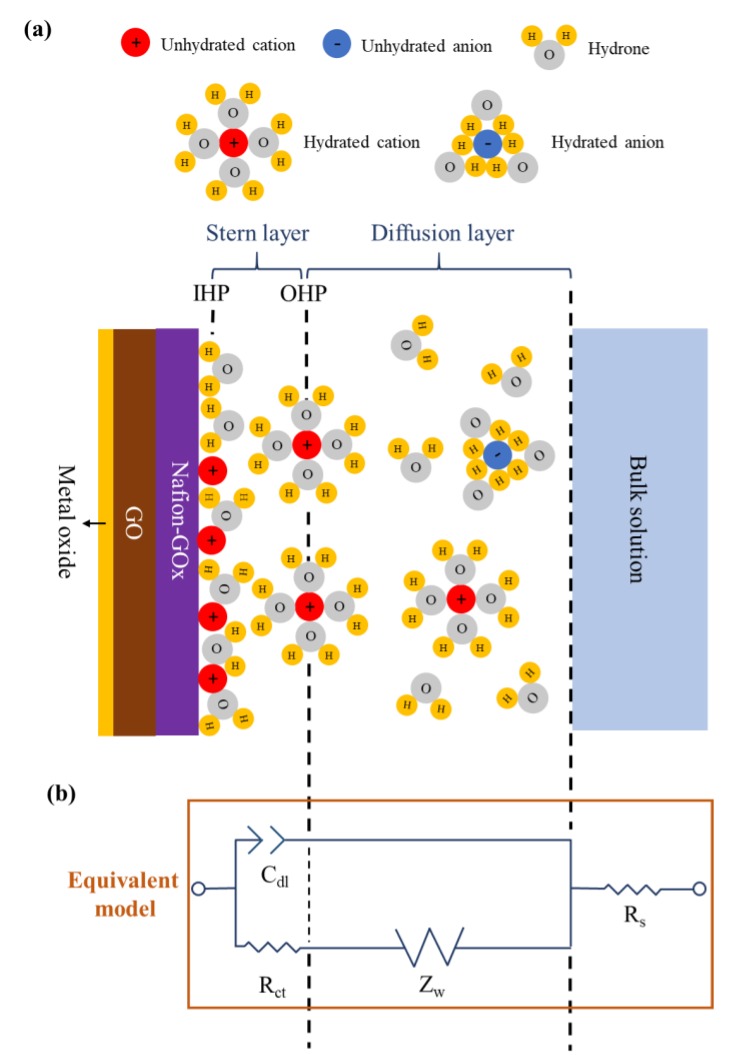
(**a**) Schematic diagram of the membrane–solution interface. (**b**) Equivalent circuit model for electrochemical impedance spectroscopy (EIS).

Electrochemical impedance spectroscopy (EIS) detects variations in electrochemical impedance within an electrochemical system by applying alternating current (AC) signals with different frequencies through an electrode, and an equivalent circuit model is used to describe the electrochemical impedance of the interface [42,43,44]. The membrane–solution interface can be described by the Stern–Grahame model [44], as shown in Figure 8a. The equivalent circuit model is shown in Figure 8b, the solution resistance (R_s_) represents the solution resistance within the working electrode and the reference electrode; the charge transfer resistance (R_ct_) represents the transfer process of charges within the electrodes and the electroactive species when the electrochemical reaction occurs; the double-layer capacitance (C_dl_) represents the electrical double layer (EDL) on the electrode surface; the Warburg impedance (Z_W_) represents the diffusion layer formed by ions in the solution. In this study, the focus of our investigation was R_ct_, i.e., the charge transfer ability of the membranes.

**Figure 9:**
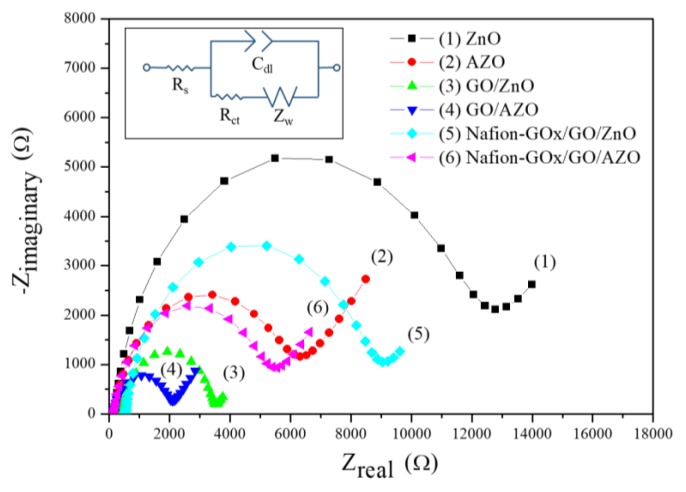
Nyquist plots of different membranes obtained by fitting of EIS.

As shown in Figure 9 and Table 2, the charge transfer resistance (R_ct_) of AZO was lower than the R_ct_ of ZnO. The results are in accordance with the previous study [16]. Next, the membranes were further modified by GO. The results show that the semicircle diameters of the GO-modified membranes (GO/ZnO and GO/AZO) were smaller than those of the non-modified membranes (ZnO and AZO), which suggests that the presence of GO can facilitate the charge transfer between the solution and membrane. This is due to the superior electrocatalytic activity of GO [45], which decreases the R_ct_ of the membrane. Finally, the GOx was immobilized on the membranes and immersed in a PBS solution (pH 7.0) containing 5 mM glucose to observe the variation of R_ct_. The R_ct_ of the membranes was greatly increased. Even if the catalytic reactions occurred, the values of R_ct_ were still higher than those obtained from bare membranes according to the results. The increase in R_ct_ can be attributed to the non-conductivities of the enzyme and Nafion. This finding indicated that the GOx was steadily immobilized on the membrane, causing the obstruction of the charge transfer [46,47].

### 3.4. Hysteresis of Potentiometric Arrayed Glucose Biosensor Based on Nafion-GOx/GO/AZO

Hysteresis is a type of non-ideal memory effect response in a potentiometric biosensor, causing a delay in potential responses [48]. The phenomenon is due to the residual potential within the solid–liquid interface arising from hydrated ions during repeated measurements, thereby resulting in the errors of response output errors. Hysteresis curves can be used to evaluate the evaluation for the stability and reversibility of a potentiometric biosensor, where V_H_ is the hysteresis voltage, which is defined as the voltage shift between initial response voltage and final response voltage [49].

**Figure 10:**
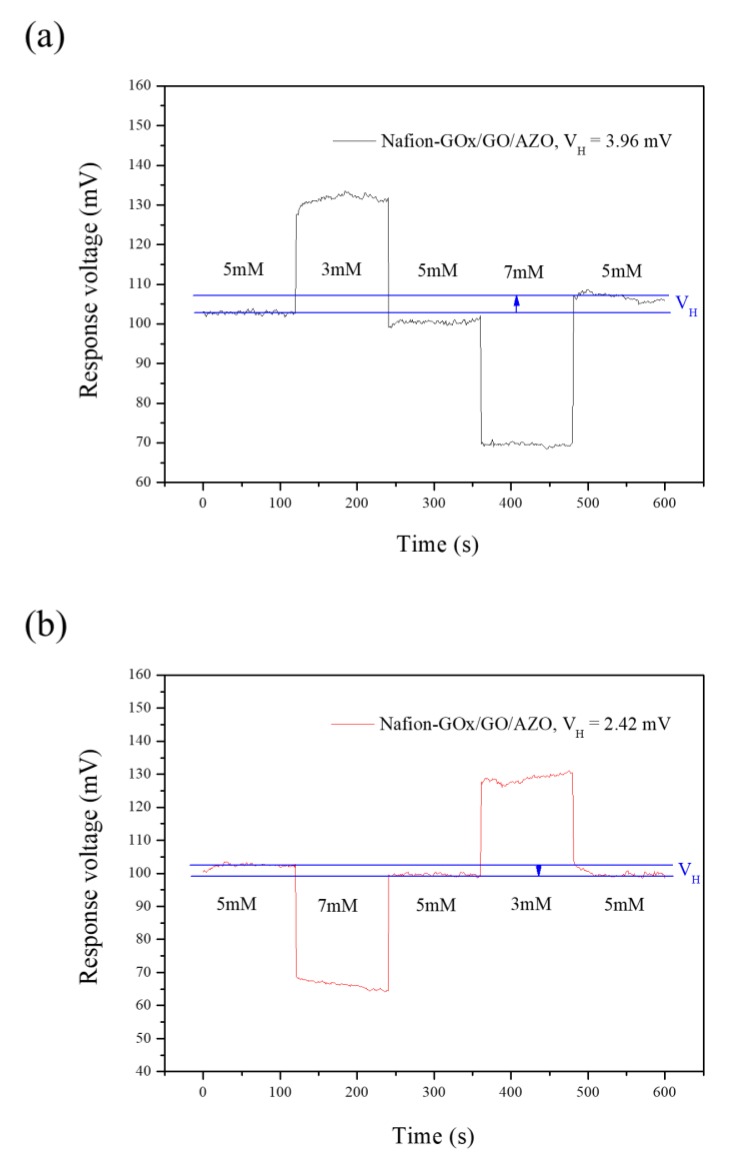
Hysteresis curves of the potentiometric arrayed glucose biosensor based on Nafion-GOx/GO/AZO across multiple cycles of (**a**) 5 mM→ 3 mM→ 5 mM→ 7 mM→ 5 mM and (**b**) in the cycle of 5 mM→ 7 mM→ 5 mM→ 3 mM→ 5 mM, respectively.

The hysteresis curves of the potentiometric arrayed glucose biosensor based on Nafion-GOx/GO/AZO were obtained across multiple cycles of different glucose concentrations (5 mM → 3 mM→ 5 mM→ 7 mM→ 5 mM and 5 mM → 7 mM→ 5 mM→ 3 mM→ 5 mM), as shown in Figure 10. The V_H_ were 3.96 mV and 2.42 mV in the forward cycle and reverse cycle, respectively. Although the local pH in the microsurrounding of electrodes was changed by the catalytic reaction of GOx, the residual potential arising from hydrated ions still caused some deviation in the response potential. The response potential did not change in accordance with expectations. This was due to the sudden change in glucose concentration, which caused a delay in the response. This phenomenon is inevitable in potentiometric biosensors. According to the V_H_ values that were obtained, the potentiometric arrayed glucose biosensor based on Nafion-GOx/GO/AZO showed good reversibility and stability.

### 3.5. Anti-Interference Ability of Potentiometric Arrayed Glucose Biosensor Based on Nafion-GOx/GO/AZO

Selectivity is a key evaluation criterion in biosensors. In this study, we selected the potential interfering substances in human blood to test the selectivity of the potentiometric glucose biosensor based on Nafion-GOx/GO/AZO, such as ascorbic acid (AA), urea, and uric acid (UA), dopamine (DA), and fructose. Substances that commonly cause interference in amperometric biosensors, such as AA and UA, can also be used to test the selectivity of potentiometric biosensors because they are highly likely to have an effect on the potential response of potentiometric biosensors [32,50,51]. Firstly, the glucose biosensor was immersed in a 0.1 M PBS solution with 5 mM glucose concentration until the response voltage was steady. The interference analytes, such as 0.06 mM AA, 5 mM urea, and 0.3 mM UA were added into the PBS solution per 60 s, sequentially. Finally, glucose was added to the same PBS solution (achieve 12 mM). The response voltage changed as expected and remained stable.

**Figure 11:**
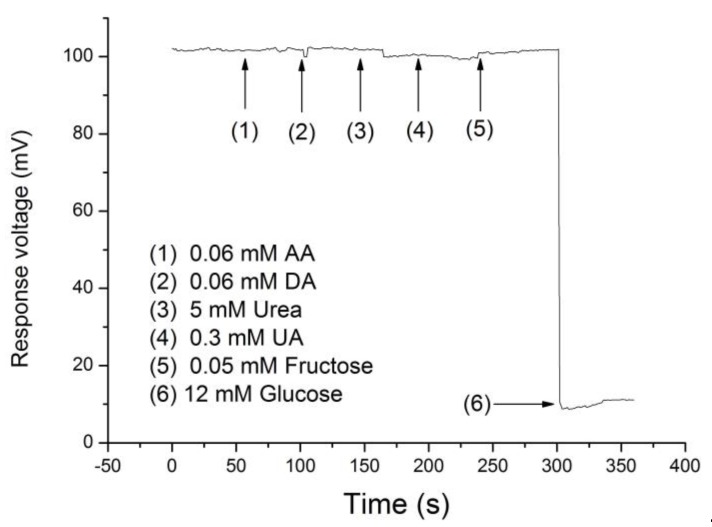
Interference effects of the potentiometric arrayed glucose biosensor based on Nafion-GOx/GO/AZO upon the addition of ascorbic acid (AA) (0.06 mM), dopamine DA (0.06 mM), urea (5 mM), uric acid (UA) (0.3 mM), fructose (0.05 mM) and glucose (12 mM).

As shown in Figure 11, the results demonstrated that the interference analytes only generated a tiny amount of noise, i.e., there was a negligible effect on the response voltage of the biosensor. Due to the high selectivity of GOx, urea did not affect the potentiometric glucose biosensor. According to a study conducted by Adeloju et al., AA had a significant influence on the glucose potentiometric biosensor [52]. However, the presence of AA and UA in our study only had a slight effect on the response signal of the biosensor. The potentiometric glucose biosensor based on Nafion-GOx/GO/AZO exhibited an excellent specificity for glucose.

### 3.6. Reproducibility of Potentiometric Arrayed Glucose Biosensor Based on Nafion-GOx/GO/AZO

**Figure 12:**
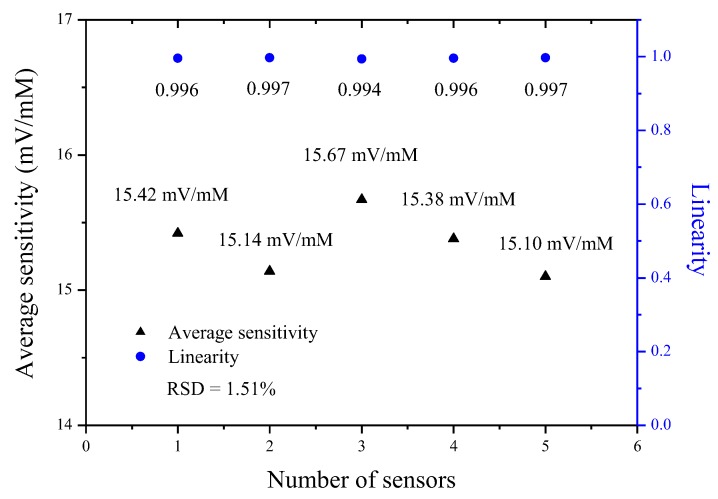
Average sensitivities and linearities of the five potentiometric arrayed glucose biosensors based on Nafion-GOx/GO/AZO over glucose concentrations ranging from 2 to 10 mM (number of sensors, *N* = 5).

In order to evaluate the reproducibility of the potentiometric arrayed glucose biosensor based on Nafion-GOx/GO/AZO, we fabricated the 15 biosensors in three batches and then selected the five sensors (number of sensors, *N* = 5), which had the optimal and similar performance among them to test (the fabrication of five biosensors in one batch). Next, the five biosensors were respectively measured in PBS solutions (pH 7.0) with glucose concentrations ranging from 2 to 10 mM, followed by calculating their average sensitivity and linearity, as shown in Figure 12 and Table 3. In Figure 12 and Table 3, it can be seen that the response voltages of biosensors were not significantly different, and the average sensitivities and linearities also showed little variation. Finally, the relative standard deviation (RSD) of the average sensitivities was determined. RSD is defined as the ratio of the standard deviation to the mean, is expressed by Equation (5):

(5) RSD= σ/μ

where σ represents the standard deviation and μ represents the mean. Herein, we used five values of the average sensitivities to find out the mean and standard deviation, as shown in Table 3. The mean of the average sensitivities was 15.34 mV/mM; the standard deviation of the average sensitivities was 0.23 mV/mM. From Equation (5), the RSD of the average sensitivities was 1.51%. These results indicated good reproducibility of the potentiometric biosensors and demonstrated that this facile fabrication was reliable.

### 3.7. Lifetime of Potentiometric Arrayed Glucose Biosensor Based on Nafion-GOx/GO/AZO

In the evaluation of a biosensor, it is crucial to establish the lifetime of the sensor, particularly as potentiometric arrayed glucose biosensors use biological materials, such as enzymes. In this study, we investigated the average sensitivity of the potentiometric glucose biosensor at different times to evaluate the lifetime. Firstly, the average sensitivity of the biosensor was recorded on the first day, which served as the datum value. Next, we measured the average sensitivities of the potentiometric arrayed glucose biosensor based on Nafion-GOx/GO/AZO every day. The data were divided by the datum value (the average sensitivity of the biosensor recorded on the 1st day), respectively, and the relative average sensitivities were subsequently obtained. Besides, the biosensor was stored at 4 °C when not in use. The lifetime of the proposed biosensor was obtained by carrying out this test, and the measuring period was a month.

**Figure 13:**
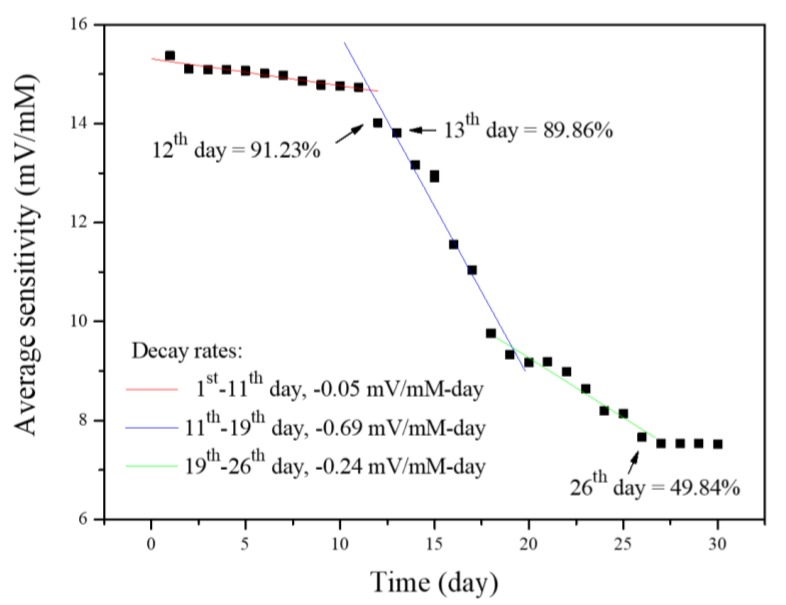
Average sensitivity variation of the potentiometric arrayed glucose biosensor based on Nafion-GOx/GO/AZO through a month.

The average sensitivity variation of the biosensor through a month is shown in Figure 13. From the 1st to the 11th day, there was a little decrease in average sensitivity; the decay rate was −0.05 mV/mM/day. From the 11th to the 19th day, it could be seen that the average sensitivity decayed obviously; the decay rate was −0.69 mV/mM/day. It was due to the large reduction of the enzyme activity and the gradual destruction of the membrane during repeated measurements. From the 19th to the 26th day, the average sensitivity still continued to decrease until the 26th day; the decay rate was −0.24 mV/mM/day. In this period, the biosensor merely relied on the residual enzymes of the membranes to remain the average sensitivity. After the 26th day, there was no variation in average sensitivity. The relative average sensitivity was about 49.84%; this value seems to be the cut-off value of the average sensitivity. The lifetime of the biosensor was determined according to the following definition; lifetime was defined as the storage or operational time required to obtain a decrease in sensitivity to 90% within the linear concentration range [52]. As shown in Figure 13, the relative average sensitivities on the 12th and the 13th day, respectively, were 91.23% and 89.86%. Therefore, the lifetime of the potentiometric arrayed glucose biosensor based on Nafion-GOx/GO/AZO was 12 days. The short lifetime was due to the restriction, which GOx caused. In addition, the lifetime of 12 days compared to similar reports [53,54] is relatively long. We also conducted the reproducibility for the sensor, as shown in Figure S2. The results showed that the average lifetime of the sensors is 12 days for the different samples, which proves that the sensors have good stability and reproducibility in this study. Biological materials inevitably shorten the lifespan of biosensors, and this is a critical challenge in the field of biosensing research. In fact, a lifetime of 12 days may be considered acceptable if this biosensor is used as a disposable device.

### 3.8. Comparisons of Glucose Biosensors

The comparisons of currently available potentiometric glucose biosensors are presented in Table 4 [8,9,10,55,56,57,58]. It can be seen in Table 4 that ZnO nanostructures applied to biosensors are prepared in two stages using both sol–gel and aqueous chemical growth deposition techniques [8,9,10]. Their difference regards the types of used electrodes and whether BSA modifies the membrane. According to the investigation by Ali et al. [8], the sensor containing BSA showed the larger linear ranges compared to the other sensor that did not contain BSA. Fulati et al. [10] reported that the sensitivity was augmented largely because the surface-to-volume ratio of ZnO NFs was higher than that of ZnO NRs. Therefore, the intracellular glucose biosensor based on a BSA-GOx/ZnO NFs/Al microelectrode showed the best sensitivity (65.2 mV/decade) and the widest linear range (500 nM to 10 mM) [10]. The potentiometric arrayed glucose biosensor based on Nafion-GOx/GO/AZO was compared to other potentiometric biosensors. However, compared to the literature [10], the experiments described here were long, and there was no further experimental analysis for the long-time measurement of the literature [10]. In this article, the stability analysis was performed in Section 3.4, and it also showed a good hysteresis voltage. Moreover, this study also showed the 12-day lifetime in Section 3.7. All biosensors are based on a single electrode such as glassy carbon electrodes (GCE) or ion-selective electrodes (ISE). Since the sensing area did not need to be determined, these single electrodes could be modified easily by the dip-coating method and aqueous chemical growth deposition techniques to grow the nanostructures. However, the proposed biosensor possessed the arrayed electrodes, and so we applied carbon nanomaterials such as GO to the modification of the electrodes by the drop-coating method, instead of the aqueous growth deposition. Although the proposed biosensor did not have a wide linear range for the detection of glucose, the sensitivity and linearity were excellent compared with other potentiometric glucose biosensors (reaching 15.44 mV/mM over a narrow glucose concentration range). In this study, the potentiometric arrayed glucose biosensor based on Nafion-GOx/GO/AZO showed good analytical performances, including an excellent average sensitivity of 15.44 mV/mM and linearity of 0.996 over a glucose concentration range (2 mM to 10 mM), a response time of 26 s, and an LOD of 1.89 mM. Moreover, we had more experiments about the stability of the biosensor, such as the temperature effect, hysteresis, and lifetime. Due to the stability for the biosensors being very important, if the biosensors were not stably sufficient, it may cause a measurement error. This is a serious problem for biosensors. According to the results, the performances of the biosensors were satisfactory in the condition of facile processes.

In this study, the potentiometric arrayed glucose biosensor based on Nafion-GOx/GO/AZO can be fabricated by using a facile method and has other advantages, including miniaturization, low cost, and feasibility of mass production. However, the analytical parameters of the proposed biosensor have room for improvement. Apart from this, the limitations induced by GOx (e.g., lifetime) should be overcome, although the glucose biosensor using GOx has excellent reproducibility and anti-interference ability. In order to develop a practical device for measuring blood glucose, further research studies will be conducted to improve the performances of the glucose biosensor. Therefore, we plan to investigate the application of nanostructures (e.g., quantum dot) or BSA to modify the enzymatic membrane [8,9], so as to enhance the linear range and response time of the biosensor. The denaturation rate of GOx must be decreased to extend the lifetime of the glucose biosensor. Finally, the measurement system and the glucose biosensor will be applied to measuring glucose in biofluid (e.g., serum) to realize a rapid, accurate, and simple detection of blood glucose.

## 4. Conclusions

We proposed a facile method for the development of the potentiometric arrayed glucose biosensor based on a PET substrate. The analytical performances were evaluated via the V-T measurement system. The average sensitivity of the potentiometric arrayed glucose biosensor based on Nafion-GOx/GO/AZO was higher than that of Nafion-GOx/GO/ZnO. The reason was attributed to the improvement of the electrical conductivity after doping Al in the ZnO lattice. Furthermore, the EIS analysis revealed that the charge transfer resistance of AZO was lower than that of ZnO. Compared to the glucose biosensors developed in recent years, the glucose biosensor based on Nafion-GOx/GO/AZO exhibited good analytical performances, such as an excellent average sensitivity of 15.44 mV/mM over a glucose concentration range (2–10 mM), a response time of 26 s, and good reproducibility. Our findings demonstrated that AZO may be a promising sensing platform, with superior performance compared with ZnO. The proposed sensor involves a relatively facile process, and the results presented herein could be useful in the development of an effective, appropriate, and easily fabricated potentiometric glucose biosensor. In the future, we hope to propose a comprehensive flexible arrayed potentiometric biosensor and realize the miniaturization of the device. Modification of the different types of the enzyme oxidase on a plurality of the working electrodes is required so that cross-comparison and integration of the data on the platform can realize the convenient detection equipment.

## Figures and Tables

**Table 1:** Sensing parameters of the potentiometric arrayed glucose biosensors over a glucose concentration ranging from 0 to 14 mM.

Membrane	Glucose Concentration (mM)	Response Voltage (Mean ± SD, mV)	Average Sensitivity (mV/mM)	Linearity
Nafion-GOx/GO/ZnO	0	118.73 ± 1.76	11.92	0.998
	2	111.64 ± 3.77		
	4	94.10 ± 4.36		
	6	67.54 ± 3.68		
	8	43.20 ± 4.16		
	10	17.88 ± 6.36		
	12	7.13 ± 5.17		
	14	1.83 ± 5.45		
Nafion-GOx/GO/AZO	0	157.02 ± 2.01	15.44	0.997
	2	145.13 ± 5.57		
	4	123.12 ± 4.80		
	6	90.32 ± 5.47		
	8	53.49 ± 6.38		
	10	25.50 ± 7.71		
	12	15.56 ± 6.58		
	14	10.72 ± 5.49		

**Table 2:** Charge transfer resistances obtained by the fitting of EIS. PBS: phosphate-buffered saline.

Membrane	Solution	R_ct_ (Ω)
ZnO	PBS	1.17 × 10^4^
AZO	PBS	5.72 × 10^3^
GO/ZnO	PBS	2.99 × 10^3^
GO/AZO	PBS	1.83 × 10^3^
Nafion-GOx/GO/ZnO	PBS (5 mM glucose)	3.53 × 10^3^
Nafion-GOx/GO/AZO	PBS (5 mM glucose)	2.31 × 10^3^

**Table 3:** Reproducibility of the five independent potentiometric arrayed glucose biosensors based on Nafion-GOx/GO/AZO over glucose concentrations ranging from 2 to 10 mM (number of sensors, *N* = 5).

Number of Sensors	Glucose Concentration (mM)	Response Voltage (Mean ± SD, mV)	Average Sensitivity (mV/mM)	Linearity
1	2	146.64 ± 3.77	15.42	0.996
	4	121.10 ± 4.36		
	6	91.54 ± 3.68		
	8	50.20 ± 4.16		
	10	27.88 ± 6.36		
2	2	145.13 ± 4.57	15.14	0.997
	4	123.12 ± 4.80		
	6	90.31 ± 5.47		
	8	53.49 ± 6.38		
	10	28.50 ± 5.71		
3	2	142.87 ± 5.46	15.67	0.994
	4	125.10 ± 4.92		
	6	89.35 ± 5.16		
	8	51.34 ± 6.01		
	10	23.07 ± 6.38		
4	2	145.25 ± 4.25	15.38	0.996
	4	124.69 ± 5.28		
	6	92.10 ± 6.16		
	8	53.46 ± 5.74		
	10	27.03 ± 5.95		
5	2	144.51 ± 6.34	15.10	0.997
	4	119.39 ± 4.31		
	6	89.37 ± 5.84		
	8	50.86 ± 3.17		
	10	27.79 ± 5.06		

**Table 4:** Potentiometric glucose biosensors based on different electrodes [8,9,10,55,56,57,58].

Electrode	Linear Range	Sensitivity	Linearity	Response Time	LOD	Ref.
Nafion-GOx/GO/AZO/Ag	2 mM to 10 mM	15.44 mV/mM	0.997	26 s	1.89 mM	This study
BSA-Nafion-GOx/ZnO NWs/Ag	0.5–1000 μM	35 mV/decade	N/A	1–4 s	N/A	[9] 2010
BSA-GOx/ZnO NFs/Al	500 nM to 10 mM	65.2 mV/decade	0.990	4 s	N/A	[10] 2010
GOx/ZnO NRs/Ag	1 µM to 10mM	2.51 mV/decade	0.980	N/A	N/A	[11] 2018
Fe_3_O_4_-GOx-Ppy/MGCE	0.5 μM t 34 mM	19.4 mV/decade	0.998	6 s	0.3 μM	[56] 2014
PAPBAOT/GCE	5–50 mM	0.58 mV/mM	0.992	200 s	0.5 mM	[57] 2013
AgNPs-GOx/Ag-ISE	0.1–3 mM	8.62 mV/decade	N/A	N/A	10 μM	[58] 2009
MIP-based on MAA	0.02–7 mM	43.7 mV/mM	0.980	N/A	N/A	[59] 2017

Note: GOx: glucose oxidase; GO: graphene oxide; AZO: aluminum-doped zinc oxide; ZnO: zinc oxide; NWs: nanowires; BSA: bovine serum albumin; NFs: nanoflakes; Ppy: polypyrrole; MGCE: magnetic glassy carbon electrode; GCE: glassy carbon electrode; PAPBAOT: poly (3-aminophenyl boronic acid-co-3-octylthiophene); MIP: molecularly imprinted polymer; MAA: methacrylic acid; NRs: nanorods.

## Supplementary Materials

The following are available online at https://www.mdpi.com/1424-8220/20/4/964/s1, Figure S1: EDX spectra of different membranes: (a) ZnO and (b) AZO, Figure S2: The reducibility test for the lifetime of the glucose biosensors, Table S1: *p*-value for response voltages intervals under different glucose concentrations.
