# Supplementary material for: A Facile Fabrication of a Potentiometric Arrayed Glucose Biosensor Based on Nafion-GOx/GO/AZO
Source: Sensors (Basel). 2020 Feb 11;20(4):964. doi: 10.3390/s20040964 (PMC7071120; doi:10.3390/s20040964)
Supplement: Supplementary file 1 [file sensors-20-00964-s001.pdf]

sensors

Supplementary Materials

(a)

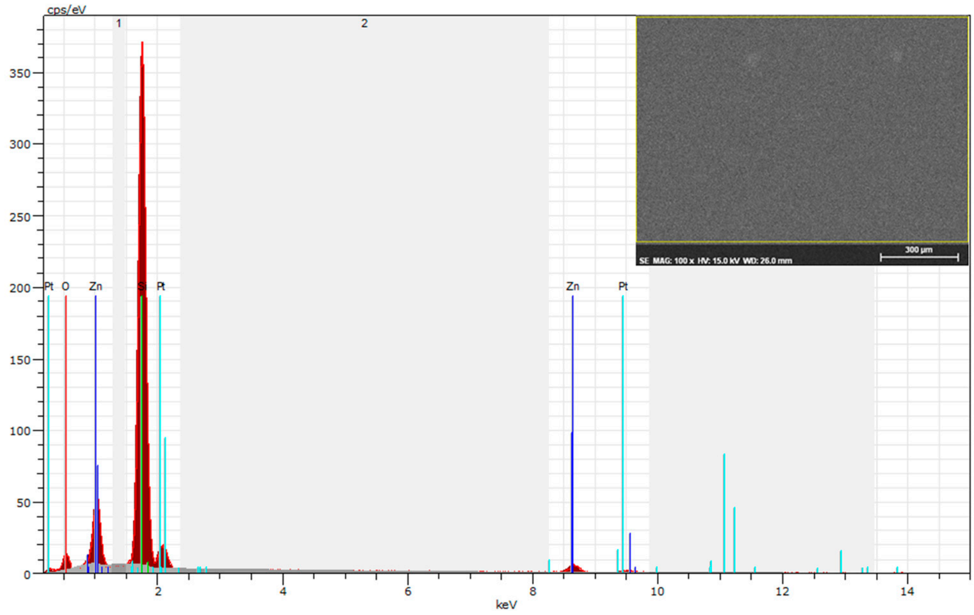

(b)

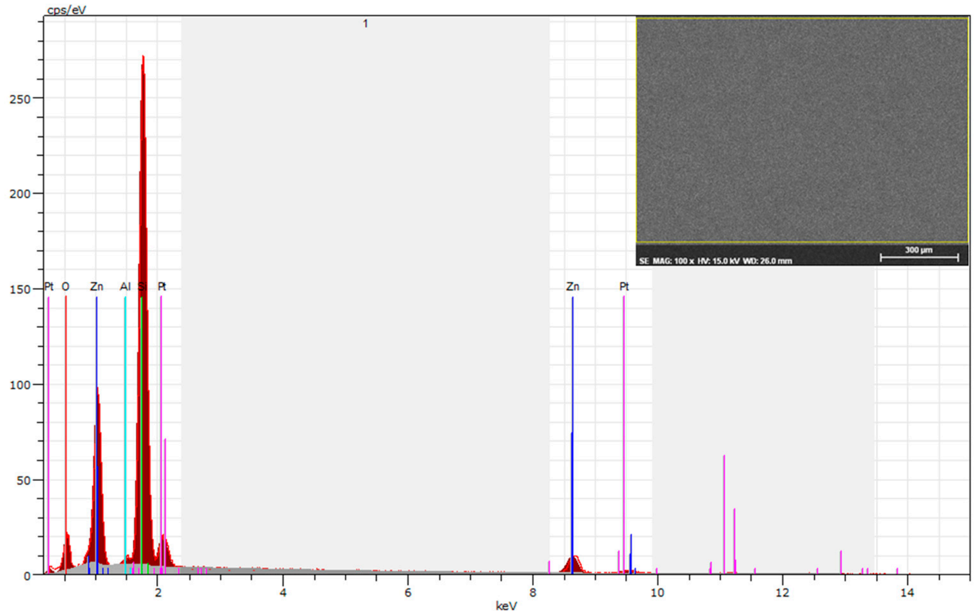

Figure S1. EDX spectra of different membranes: (**a**) ZnO and (**b**) AZO.

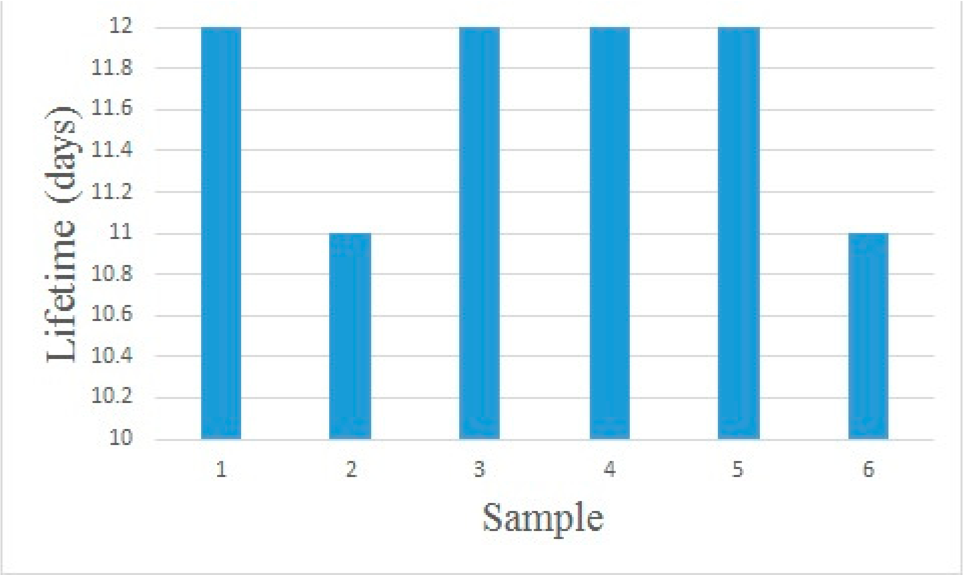

Figure S2. The reducibility test for the lifetime of the glucose biosensors.

Table S1. *p*-value for response voltages intervals under different glucose concentrations.

**Membrane**	**Interval of Concentration**	**N (6 windows)**	**Degree of freedom**	***p*-Value (Two-Tailed Tests)**	**Significance**
Nafion-GOx/GO/ZnO	(0, 2)	6	2	1.92×10^{-3}	**
	(2, 4)			2.17×10^{-5}	***
	(4, 6)			4.72×10^{-7}	***
	(6, 8)			8.27×10^{-7}	***
	(8, 10)			9.87×10^{-6}	***
	(10, 12)			9.32×10^{-3}	**
	(12, 14)			1.14×10^{-6}	N/A
Nafion-GOx/GO/AZO	(0, 2)	6	2	6.06×10^{-4}	***
	(2, 4)			2.51×10^{-5}	***
	(4, 6)			6.37×10^{-7}	***
	(6, 8)			8.32×10^{-7}	***
	(8, 10)			4.47×10^{-5}	***
	(10, 12)			3.72×10^{-2}	*
	(12, 14)			1.97×10^{-1}	N/A
